# Cytotoxic effect of silorane and methacrylate based composites on the human dental pulp stem cells and fibroblasts

**DOI:** 10.4317/medoral.19340

**Published:** 2014-03-08

**Authors:** Fereshteh Shafiei, Maryam S. Tavangar, Mahboobeh Razmkhah, Armin Attar, Ali A. Alavi

**Affiliations:** 1 Operative Dentistry Department, Dental Faculty, Shiraz University of Medical Sciences, Shiraz, Iran; 2 Biomaterial Research center, Dental Faculty, Shiraz University of Medical Sciences, Shiraz, Iran; 3 Shiraz Institute for Cancer Research, School of Medicine, Shiraz University of Medical Sciences, Shiraz, Iran; 4 Student Research Committee, Shiraz University of Medical Sciences, Shiraz, Iran; 5 Cell and Molecular Medicine Research Club, Shiraz University of Medical Sciences, Shiraz, Iran

## Abstract

Objectives: The aim of this study was to compare the cytotoxic effect of a methacrylate-based and a silorane-based composite on the human dental pulp stem cells (DPSCs) versus human dental pulp fibroblasts (DPFs). Study Design: Samples of the Filtek Z250 and P90 were polymerized and immersed in the culture medium to obtain extracts after incubation for one, seven and 14 days. Magnetic cell sorting based on the CD146 expression was performed to purify DPSCs and DPFs. After incubation of both cells with the extracts, cytotoxicity was determined using the MTT test. Results: For the extracts of first and seventh day, both composites showed significantly lower cytotoxicity on DPSCs than DPFs (p=0.003). In addition, there was a significant difference in the time-group interaction of both materials indicating different cytotoxic behaviours (p=0.014). In contrast to Z250, exposure to the 14th day extract of P90 resulted in higher cell viability compared to that of day seven. Conclusions: DPSCs are less susceptible to the cytotoxic effect of the composites than DPFs. Compared to Z250, the cytotoxic effect of silorane-based composite decreases as the time passes on. This difference should be considered, particularly in deep cavities, in order to preserve the regenerative capacity of the pulp.

** Key words:**Composite resins, Dental pulp, Mesenchymal Stromal Cells, Silorane, Toxicology.

## Introduction

Composite resin materials are widely used in various applications in routine dental restorative procedures. Traditionally, most of composite resins are methacrylate-based and their polymerization initiates by the free-radical mechanism (1). The shrinkage stress generated during polymerization of methacrylate-based composites is responsible for major clinical disadvantages including cuspal deflection, marginal gap, micro-leakage, postoperative sensitivity and recurrent caries (2). In order to overcome this problem, a new monomer system based on silorane has been introduced. Siloxane and oxirane molecules are the structural moieties of this monomer. Siloxane imparts the hydrophobic properties of silorane monomers while oxirane is responsible for its low shrinkage during polymerization. The mechanism of this polymerization relies on the ring opening cationic reactions (3).

Despite the significant improvements in the physical and aesthetic properties of modern resin-based composites, there are some concerns about their biocompatibility (4). Many in vitro studies have shown that substances released from the composite resins due to resin degradation or incomplete polymerization can diffuse through dentin and reach the pulp tissue (5). These substances are able to affect the vitality and regenerative capacities of the pulp (6). The regenerative capacity of the pulp tissue has been attributed to the residing stem cells (7). Pulp-derived stem cells are a population of undifferentiated cells with self-renewability, colony forming capacity and ability to differentiate into several cell lineages. “Stem cells of human exfoliated deciduous teeth (SHED)” and permanent tooth-derived “dental pulp stem cells (DPSCs)” are current representatives of pulp-derived stem cells (8). They remain quiescent until deep cavity preparation or severe injuries to the pulp occur. The resultant odontoblasts layer destruction leads to proliferation and migration of stem cells to the injury site and their differentiation into odontoblast-like cells. These odontoblast-like cells secrete reparative dentine as a protective barrier in response to pulp injury (7). Therefore, preserving pulp-derived stem cells following a restorative procedure can play an important role in maintaining the regenerative capacity and recovering the pulp vitality.

Cytotoxicity of composite resins has been widely investigated in deep cavities or on various pulp cells such as human pulp fibro-blasts (9), immortalized odontoblast-cell line (10) and human-transformed pulp-derived cells (6). However, the cytotoxic effect of these compounds has not been evaluated on DPSCs yet. The aim of this study is to investigate the viability of the DPSCs and dental pulp derived fibroblasts (DPFs), as terminally differentiated cells, after exposure to methacrylate and silorane-based composites.

## Material and Methods

-Sample preparation

Two currently used composite resins including Filtek Z 250 and Filtek P 90 (3M ESPE, St Paul, MN, USA) in the same shade (A3) were used in this study ( Table 1). To prepare disc-shaped samples, Teflon moulds (two mm in thickness and four mm in diameter) were placed on the glass plate and filled with composites. Then, the samples were polymerized for 20 seconds with the Radii Plus LED (Light Emitting Diodes) (SDI, Victoria, Australia) using standard mode (1500 mW/cm2). The composites were covered with a Mylar strips (Moyco Union Broach, York, USA) during light activation. Eight samples of each composite were prepared in the same way. For each material, four out of eight samples were used to analyze the cytotoxicity of materials on the dental pulp stem cell and the others were tested on dental pulp fibroblast. Each sample was immediately immersed in 300μl DMEM culture medium (Dulbecco’s Modified Eagle Medium, Gibco/ Invitrogen, Carlsbad, CA, USA) and stored in the dark at 37◦C. The ratio of the sample surface area to the volume of the solution was 1.7cm2/ml which is within the recommended range (0.5–6.0cm2/ml) by ISO (International Organization for Standardization) (11,12). The extracts from each sample were collected in the test-tubes after incubations for one, seven and 14 days and each extract was used separately for proceeding steps.

**Table 1:**
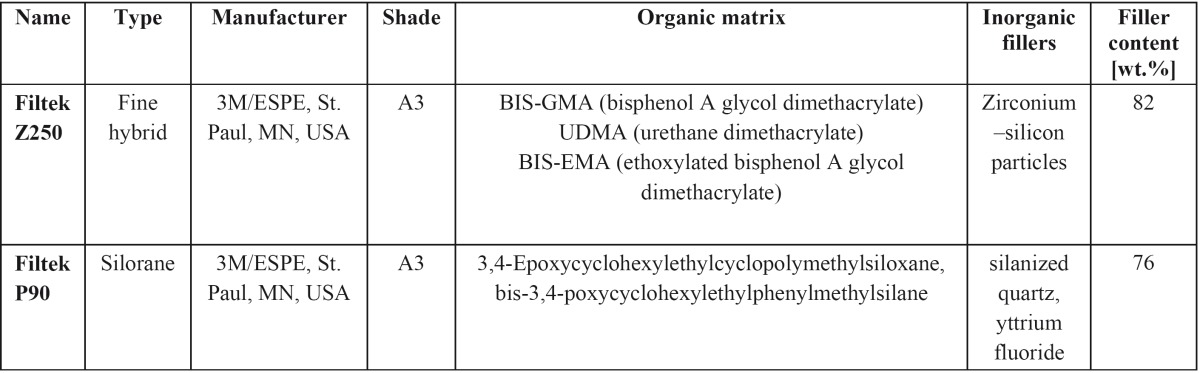
Composites.

Preparation of single cell suspension from human pulp

Human third molars were collected from young adults aged 20-25 years. All the patients gave their written consent before enrolment in the study. This study was approved by the local ethics committee. Specimens were cut from around the cement-enamel junction using a fissure burs. Pulp tissue was then gently removed from the chambers and digested using an enzyme solution consisting of three mg/ml collagenase type I and four mg/ml dispase II (both from Sigma, St. Louis, MO, USA) for 30-60 minutes at 37 °C. The digest was centrifuged at 1200 rpm for 5 minutes. The pellets were then suspended for the next steps.

-Culturing the isolated cells

The single cell suspensions were plated in DMEM supplemented with 4 mM GlutaMAX, 100 U/mL penicillin, 100 µg/mL streptomycin and 20% FBS (All from Gibco/ Invitrogen, Carlsbad, CA, USA). Cells were cultured at 37ºC in 5% CO2 and 90% humidity. Culture media were changed twice weekly until the flask reached 80% confluency. Then the cells were released with tryp-sin–EDTA (Gibco) and sub-cultured. These cells were passaged three times before undergoing CD146 magnetic cell sorting.

-Magnetic cell sorting of the cells

Magnetic cell sorting was designed in order to purify stem cells from fibroblasts with separation of CD146 positive stem cells from CD146 negative fibroblasts (13). The cells from the 3rd passage underwent dead cell elimination using Dead cell removal kit (Miltenyi Biotec GmbH, Bergisch Gladbach, Germany) to decrease the chance of non-specific cell bindings of antibodies. Cells were firstly labeled with FITC-conjugated CD146 antibody (BD Biosciences, San Jose, CA, USA). Then the target cells were labeled with the anti-FITC microbeads (Miltenyi Biotec). The cell suspension underwent magnetic cell sorting regarding the manufacturer’s instructions. The negative and positive isolated cells underwent some experiments to approve stemness including colony forming assay, differentiations and cell surface antigen markers analysis with flowcytometry as is explained below.

-Colony forming unit assay

To assess the efficiency of single cell derived colony formation (Colony Forming Unit Fibroblast assay [CFU-F]), CD146 positive and negative portions were seeded into six well plates with the density of 25000 cells per well. Single cell derived colonies were defined as those units with more than 50 cells. The number of colonies was counted one day before the colonies were merged or as late as 14 days of culture.

-Differentiation 

For adipogenic differentiation, the cells after magnetic cell sorting were cultured in MesenCult medium supplemented with 10% Adipogenic Stimulatory Supplements (both from Stem Cell Technologies Inc, Vancouver, BC, Canada) regarding the manufacturer’s guidelines. To approve the differentiation, the cells were analyzed with Oil-red O staining and underwent RT-PCR. For osteogenic differentiation, the cells after magnetic cell sorting were cultured in NH-osteoDiff Medium (Miltenyi Biotec) according to the manufacturer’s guidelines. To approve the differentiation, the cells were stained with Alizarin red and underwent RT-PCR.

-Reverse Transcription-Polymerase Chain Reaction

RT-PCR was performed to determine the expression of adipocyte and osteoblast marker genes in induced cells. To assess the osteogenic differentiation, RT-PCR for detection of osteopontin and col-1α1 (pro-alpha1 chains of type I collagen) mRNA was performed. To assess the differentiation into adipocytes, RT-PCR was performed for detection of PPAR-γ2 (peroxisome prolifera-tor-activated receptor gamma, transcript variant 2) and aP2 (adipocyte protein 2) (8). The sequences of the primers are shown in table 2.

Total RNA was extracted with TriPure Isolation Reagent (Roche Applied Science, Mannheim, Germany). The RNAs were re-verse-transcribed to first strand complementary DNA (cDNA) by using the Sensiscript® Reverse Transcription kit (Qiagen, Hilden, Germany). RT-PCR cycles were performed in a reaction mixture consisting of reverse and forward primers (Metabion international AG, Martinsried, Germany) ( Table 2), dNTP mixture, 10x PCR buffer, MgCl2, Taq DNA polymerase (Fermentas, Life Science, EU), and distilled water. PCR products were analyzed by two percent agarose gel electrophoresis and visualized by ethidium bromide staining.

**Table 2:**
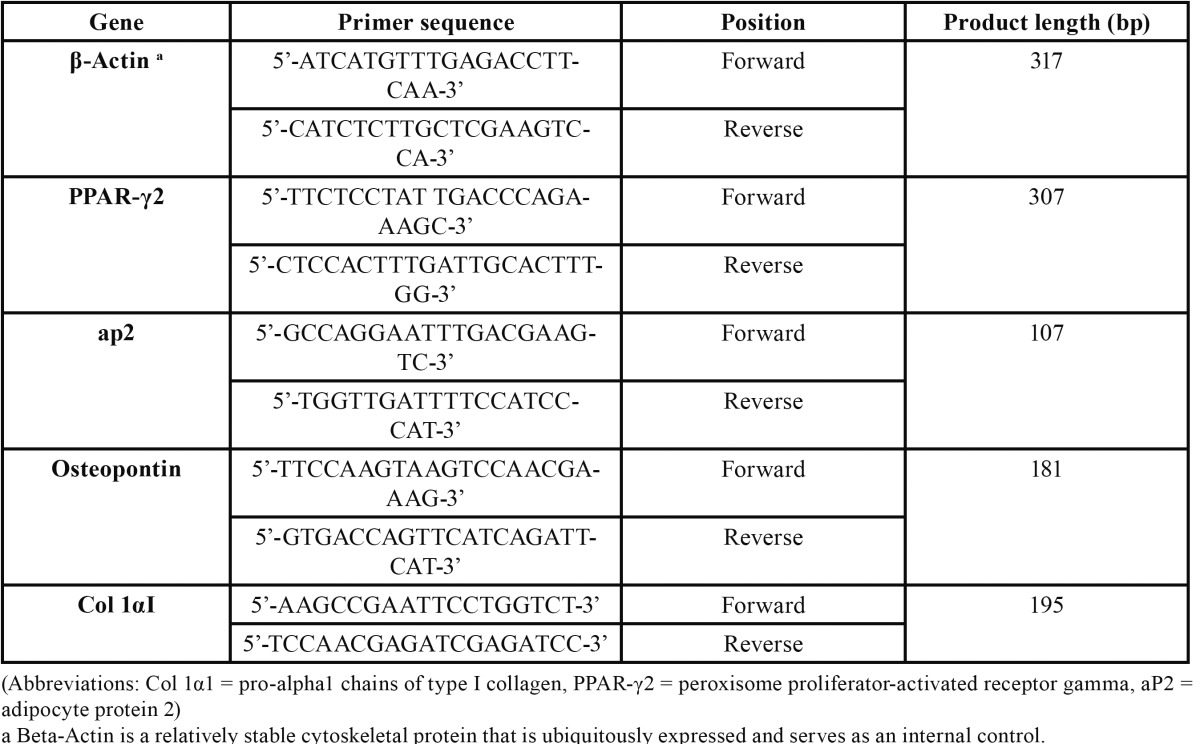
PCR primer sequences and their amplified product size.

-Flow cytometry

To analyze the cell surface antigen expressions, the cells after CD146 magnetic cell sorting were used. The isolated cells were incubated 10-30 minutes in dark environment with the following anti-human antibodies: CD90–Allophycocyanin (APC), CD34-FITC and CD45-Peridinin chlorophyll protein (PerCP) (Miltenyi Biotec), CD14-FITC, CD166- Phycoerythrin (PE), CD44-FITC, CD146-FITC, HLA-DR-PerCP, CD73-PE (BD Biosciences) and STRO1-pure (With secondary anti-IgM FITC con-jugated IgG, Santa Cruz Biotechnology Inc., Santa Cruz, CA, USA). Isotype-matched irrelevant monoclonal antibodies (Mouse IgG1-PE, IgG1-APC, IgG2a-FITC (AbD Serotec, Kidlington, Oxford, UK), IgG2a-PerCP, IgG2b-FITC, IgG2a-PE (Miltenyi Biotec)) were used to exclude non specific-staining of the cells. Flow cytometric analysis was performed on a FACS Calibur instrument (BD Biosciences, USA), using the Cell quest as data acquisition software. The WinMDI 2.8 software was used for data analyses.

-Thiazolyl Blue Tetrazolium Bromide (MTT) assay

DPFs and DPSCs were seeded into 96-well micro-plates with a cell density of approximately 10,000 cells per well. We topped up each well with 100 μl of composite extracts in order to check their cytotoxicity. Eight parallel test batches with each composite and 8 control batches with DMEM were obtained. The cell cultures were incubated with the test substances for 24 hrs at 37 °C and 5% CO2.

Then the viability of DPFs and DPSCs was determined using the MTT assay. For this purpose, the culture media were com-pletely removed and 150 μl of 0.1% MTT solution in complete culture medium was added. Cells were incubated for five hours at 37 °C in a 5% humidified CO2 incubator. After incubation, the formazan crystals were dissolved in 150 µl Dimethyl Sulfoxide (DMSO) and the absorbance was read at 490 nm after 24 hours. The cell viability was calculated regarding the following equation.

Cell viability [%] = 100×OD mean of test groups/OD mean of control groups

-Statistical analysis

The data were analyzed in SPSS software version 15.0 for windows (IBM, USA). Graphpad Prism five (Inc; San Diego CA, USA) was used for graphical presentation of data. The significance level was tested by analysis of variance with repeated measurements, and paired t-test. p<0.05 was determined as the significance level.

## Results

-Culture Characteristics

**Figure 1:**
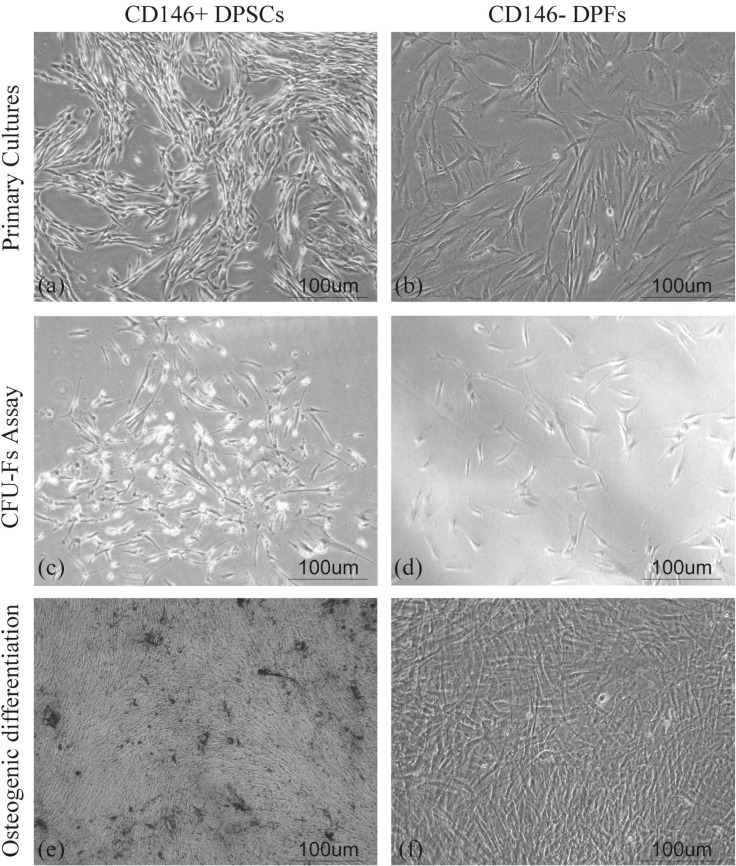
Morphology of cells within cultures: a) Typical fusiform fibroblast like appearance of cells from CD146 positive and b) CD146 negative cultures. c) A single cell derived colony from CD146 positive derived cells formed within CFU-Fibroblast assay. d) CD146 negative cells could not from any colonies. e) CD146 positive cells could perform matrix calcium deposition during osteogenic differentiation and f) CD146 negative cells failed to do so. (Abbreviations: DPSC=Dental Pulp Stem Cell, DPF=Dental Pulp Fibroblast, CFU-F=Colony Forming Unit Fibroblast).

Two days after the initial seeding, the pulp derived cells as well as CD146 positive and negative cultured cells were attached to the plates and became confluent within 12-21 days with a typical fusiform fibroblast like appearance (Fig. 1).

-Colonogenic efficacy

To assess single cell derived colony formation (CFU-F assay), as a marker of stemness and self-renewability, only colonies with more than 50 cells were considered in colony enumeration (Fig. 1). Every 10,000 CD146 positive cells could form 96±18.7 CFU-Fs. On the other hand, CD146 negative portion did not show any colonies. This difference was significant (P<0.001).

-Differentiation assay

Differentiation was done in order to confirm multi-potentiality of the cells. Negative and positive portions of the cells were cul-tured in differentiation media for up to four weeks. CD146 positive cells differentiation to adipocytes was confirmed through morphological changes and related staining. These cells could form vacuoles that increased in number and size with time and all of them were stained by Oil red O. The expression of PPAR-γ2 and aP2 mRNAs, as shown by RT-PCR, further confirmed this differentiation. The morphologic evidence of differentiation of CD146 positive cells to osteoblasts was matrix depositions around the cells (Fig. 1). Mineralization was documented by alizarin red staining. The differentiated cells typically expressed osteopontin and col-1α1, as was shown by RT-PCR. CD146 negative cells could not do any of the above (Fig. 1).

.Flow cytometric results

After CD146 cell sorting, the purity of the isolation was shown to be 91.75± 2.37%. The negative portion of the cells resulting from magnetic cell sorting was only 2.89± 1.2% positive for CD146. Flow cytometric analysis of both positive and negative portions showed that the cells were positive for mesenchymal markers such as CD44, CD166, CD90 and CD73, and were negative for surface molecules CD14, CD34, CD45 and HLA-DR. The CD146+ portion showed to be uniformly positive for STRO-1 (89.97± 4.76%) but the negative portion showed a partial expression of STRO-1 (28.93± 6.5%). The complete results from flow cytometric analysis are displayed in  Table 3.

**Table 3:**
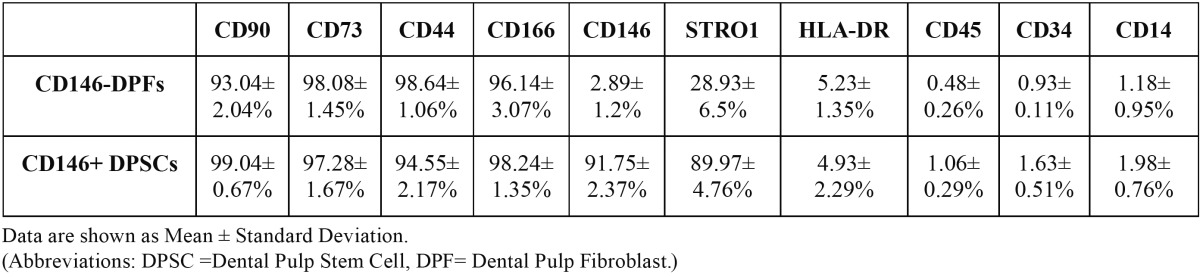
Percentage of cell surface antigens on the analyzed cells.

-Cytotoxicity

**Figure 2:**
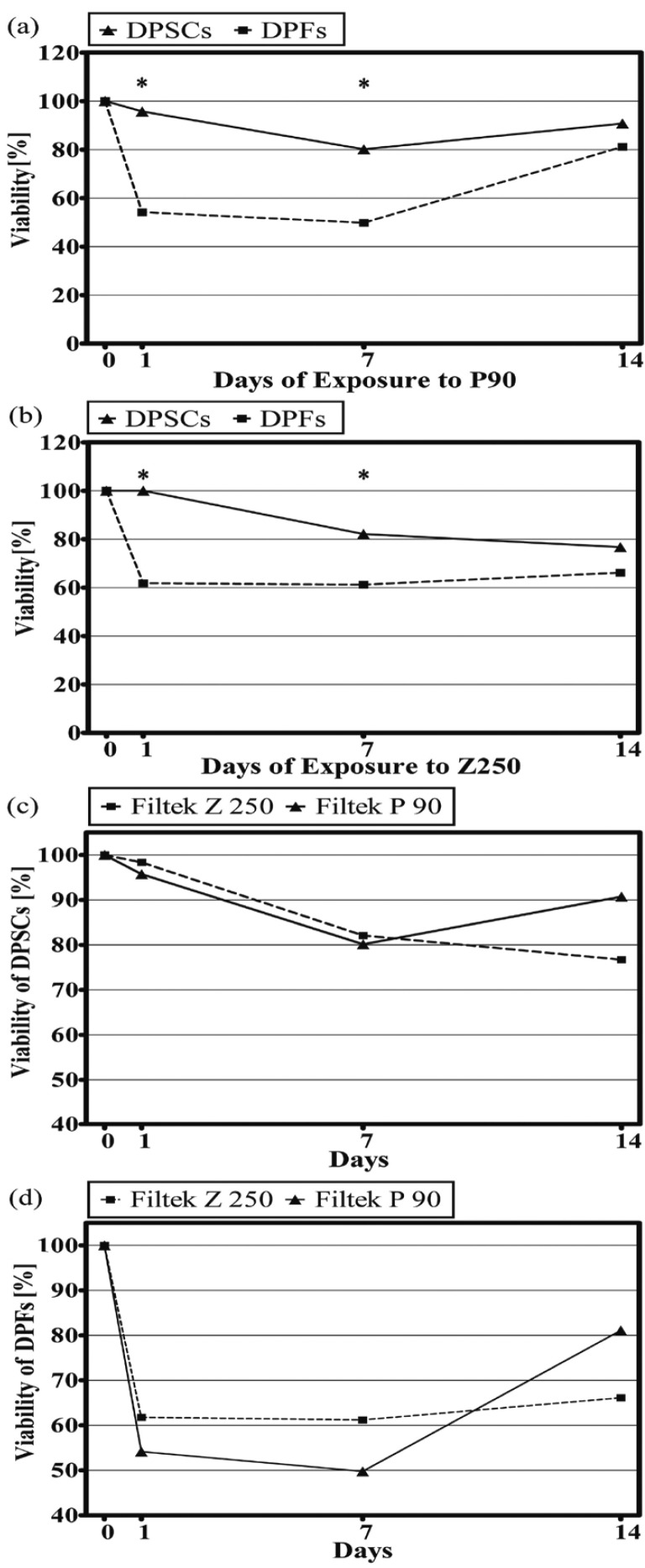
Cell viability after incubation with composite extracts: a) viability of DPSCs and DPFs after incubation with the extracts of P90; b) viability of DPSCs and DPFs after incubation;*p<0.05; c) DPSCs viability after incubation with the extracts of P90 and Z250 with significant difference in the time- group interaction between Z250 and P90; d) DPFs viability after incubation with the extracts of P90 and Z250 with significant difference in the time-group interaction between Z250 and P90.

Results of the MTT assay for the cytotoxicity of the Filtek P90 composite showed significantly higher vitality in DPSCs after exposure to the extract from the first and seventh days than that of DPFs (*p*=0.021 and *p*=0.003, respectively) (Fig. 2). Similarly, DPSCs exposed to Filtek Z250 showed higher viability than DPFs after incubation with the extract of the first (*p*=0.029) and seventh days (*p*=0.003) with no significant difference for the extract of the 14th day (Fig. 2).

In addition, there was a significant difference in the time-group interaction of Z250 and P90 with DPSCs, indicating different cytotoxic behaviors of the two materials (*p*=0.014). The exposure of DPSCs to Filtek P90 resulted in an increase in the cell viability for the extract of day 14 compared to day seven. Conversely, for Z250, exposure to the extract of the 14th day further diminished the cell viability (Fig. 2). Almost similar results were noticed in the analysis of the time-group interaction of the composites with DPFs (*p*=0.035). After a marked reduction in the viability of the cells incubated with the extract obtained from the first day, the seventh day extracts led to nearly constant values of cytotoxicity. Compared to the seventh day, higher cell viability was observed after exposure to the extract of the 14th day which was more prominent for the P90 compared to the Z250 (Fig. 2).

## Discussion

In the present study, we assessed the viability of DPSCs and DPFs after exposure to Filtek Z 250 and FiltekP90 representing two widely used composite resins based on different monomer compositions. To this end, DPFs and DPSCs were isolated from the culture of the pulp derived cells using magnetic cell sorting with respect to CD146 expression. The stemness of positive portion (DPSCs) was confirmed by the ability to form colonies and differentiate to adipocytes and osteoblasts. The negative counterpart (DPFs) failed to exhibit any of the mentioned stemness features. DPFs were used in this survey as a model of terminally differentiated cell which from a large population in the pulp tissue.

Fibroblasts are plastic adherent cells that contribute to synthesis and remodeling of extracellular matrix in tissues. In contrast to stem cells, these cells don’t have the ability to differentiate into other cell types (14). With respect to the definition by international society of stem cell therapy, mesenchymal stem cells (MSCs) are currently defined as plastic adherent fibroblast-like cells with the capacity to differentiate into at least adipocytes and osteoblasts. These cells should be positive for markers such as CD73, CD105 and CD90 while not expressing CD45, CD34 and CD14 (15). Since DPSCs share many common features with MSCs, they are categorized as a type of MSCs and the above criteria are also applied for their definition, considering that these cells are more committed to odontogenic development (8).

It is well known that the routine cultures of DPSCs, which are achieved simply by direct culturing of tissue derived single cell suspensions, are very heterogeneous(8). One of the explanations for this phenomenon can be the contamination of stem cells with mature cells including fibroblasts (16). Recently, it has been mentioned that some parameters including plastic adherence, spindle-like morphology or cell surface markers such as CD14, CD34, CD44, CD45, CD73 and CD105 that are commonly used for defining stem cells, cannot distinguish these cells from fibroblasts (17). New investigations to find discriminating factors have been recently performed. Alt et al. showed that colony-forming capacity could serve this purpose. Another study analyzing wider panels of cell surface antigen markers showed that among all the studied antigens only CD146 expression was restricted to the stem cells and absent on the fibroblasts (13). Furthermore, it is indicated that the entire colony forming units are limited to the CD146 positive cells (18). These findings prove that CDl46 expression can discriminate true stem cells from fibroblasts. Accordingly, we used CD146 isolation approach to purify both stem cells and fibroblasts from a pulp tissue and we clearly revealed that only the CD146 positive portion of the cells could form colonies and differentiate into adipocytes or osteoblasts while the CD146 negative portion failed to do so. To the best of our knowledge, this approach for simultaneous purification of both DPSCs and DPFs from a routine heterogeneous mixed culture of pulp derived cells has not been reported before.

In the current study, the analysis of the cytotoxicity of the extracts showed that the viability of DPSCs is significantly higher than DPFs after one and seven days of exposure to the extracts of both Filtek Z250 and Filtek P90. This difference can be attributed to higher resistance of stem cell to cytotoxic agents than the fibroblasts. Currently, our knowledge about the resistance of mesenchymal stem cells, particularly DPSCs, to cytotoxic agents is limited. Sauer et al. showed that BM-MSCs express detectable levels of a protein called ATP7B which protects them from toxic copper (19). The resistance to cytotoxic agents has been widely investigated in hematopoietic stem cells (HSCs). In the hematopoietic hierarchy, the resistance of cells to cytotoxic agents decreases as the stemness of cells diminishes and a more mature phenotype appears. This phenomenon has been attributed to expression of some proteins from a superfamily named ATP-binding cassette (ABC) transporters. High expression of some ABC transporters contributes to protecting cells from cytotoxic drugs through an ATP-dependent drug efflux mechanism. In the primary investigations, a correlation has been found between an undifferentiated state in normal hematopoietic cells and two members of the ABC transporter genes (ABCB1and ABCG2). Subsequent surveys depicted that ABCG1, ABCA1, ABCB1, ABCC1, ABCD4 and ABCB2 genes were predominantly expressed in the hematopoietic stem cells and down-regulation of these genes occurs upon differentiation into a progenitor state (20). The biology of differential cytotoxic agent resistance in DPSCs in comparison to DPFs is not clear but may be related to the expression of drug resistance proteins. However, further studies are required to clarify the mechanism of this resistance to the cytotoxic agents.

According to the obtained results, methacrylate-based and silorane-based composites exhibited different cytotoxic behaviors particularly after the first week of experiment. For silorane-based composite, the trend of reduction in the cytotoxicity was detected in the both cell types whereas no significant reduction was found in the cytotoxic effect of methacrylate-based on the DPFs and even an increase in the cytotoxicity was noticed in the DPSCs. This finding is in accordance with that of a previous in vitro study reporting that the cytotoxicity of Hermes, a silorane-based material, reduced markedly with time, but no improvement in the cell viability was observed for a methacrylate-based composite, even after eight weeks (21). The different cytotoxic profiles of the silorane-based composite may be attributed to the hydrolytic stability of this material (6). Palin et al. showed that compared to methacrylate-based composite (Filtek Z250), silorane-based composite exhibited lower solubility, water sorption and diffusion coefficient following short- and medium-term immersion periods (22). These hydrophobic properties diminish the release of unpolymerized monomers (3). In addition, it has been suggested that the reduction in the cytotoxicity of silorane-based composite is caused by the lower amount of residual monomers after polymerization procedure (21). However, there is a contradicting report which has described a lower degree of conversion for this material in comparison with methacrylate-based composites (Filtek Z250) (23). Despite the findings of previous in vitro studies, results from an in vivo study showed that compared to silorane, applying a methacrylate-based composite in the deep dentin cavities caused no more adverse pulpal and periapical reactions (24).

In this in vitro study, the samples were polymerized using the LED unit with the relatively high intensity (1500 mW/cm2) at the distance of one mm from the resin surface. This distance (one mm) is commonly recommended for the position of the light curing appliance tip. However, during clinical procedures, factors such as configuration and depth of cavity, cuspal tips and the position of the tooth in the arch can interfere with the proximity of the appliance tip to the composite particularly in the first increment of resin. It was shown that as the distance from the curing tip increases, the light intensity and polymerization depth diminishes (25). The lower degree of conversion, caused by the incomplete polymerization, can increase the cytotoxic effect of composite resins (4). Therefore, more variability on cytotoxic effect of these composites may occur in the clinical situation.

Although at present there is no report about the cytotoxic effect of dental materials on the pulp derived stem cells, few studies have investigated other biological effect of the resin on these cells. For an example, Bakopoulou et al. depicted that 2-hydroxy-ethyl-methacrylate (HEMA) and triethylene-glycoldimethacrylate (TEGDMA) had a negative significant effect on the odontogenic differentiation potential of SHEDs, which might disturb pulp tissue repair (26). It is not completely clear that how the residual monomers interact with the pulp cells, but they may impose their cytotoxic effects via apoptosis in pulp cells, induce genotoxic effects and delay the cell cycle. They also influence the response of cells of the innate immune system. TEGDMA and HEMA can inhibit the expression of CD14 in macrophages (27) and Bisphenol-A can alter macrophage adhesion and the process of inflammation (28).

Considering the interference of resinous monomers with differentiation of stem cells into odontoblasts producing a reparative dentin, the concerns have been raised regarding the safety of some clinical procedures such as direct pulp capping or placement of composites restoration in deep cavities with methacrylate-based materials (26). When the residual dentin thickness is below 1mm or acid etching is performed, diffusion of resinous monomers through dentinal tubules markedly increases. On the other hand, in the low depth cavities minor histological reactions have been shown in the pulp as a result of dental material application (4). Therefore, further investigations, such as dentin barrier test, are required to mimic the clinical condition more closely.

Within the limitation of this in vitro study and based on the obtained results, compared to the DPFs, DPSCs are less susceptible to the cytotoxic effect of FiltekP90 and FiltekZ250. Furthermore, it can be concluded that compared to FiltekZ250, the cytotoxic effect of silorane-based composite on DPSCs and DPFs decreases as the time passes. When the resin composites are applied in deep cavities with limited residual dentin thickness, this difference in the cytotoxic behavior should be considered in order to preserve the regenerative capacity of the pulp cells.
